# Erythrocyte Glutathione/Redox Balance and Serum Thiol/Disulfide Homeostasis in Hepatitis B and Hepatocellular Carcinoma

**DOI:** 10.7759/cureus.96763

**Published:** 2025-11-13

**Authors:** Nuray Uremis, Kadir Gisi, Teoman Sakalar, Fatma Inanc Tolun

**Affiliations:** 1 Department of Medical Biochemistry, Kahramanmaras Sutcu Imam University, Kahramanmaras, TUR; 2 Department of Gastroenterology, Kahramanmaras Sutcu Imam University, Kahramanmaras, TUR; 3 Department of Medical Oncology, Kahramanmaras Sutcu Imam University, Kahramanmaras, TUR

**Keywords:** antioxidant, glutathione, hepatitis b, hepatocellular carcinoma, oxidative stress, thiol

## Abstract

Background

Oxidative stress and imbalance in thiol-disulfide homeostasis play a crucial role in the pathogenesis and progression of liver-related diseases. This study aimed to determine the thiol-based redox system and glutathione homeostasis by evaluating erythrocyte glutathione (GSH), superoxide dismutase (SOD), total antioxidant status (TAS), total oxidant status (TOS), and malondialdehyde (MDA) levels and serum thiol/disulfide and GSH parameters in patients with hepatitis B virus (HBV) and hepatocellular carcinoma (HCC).

Methodology

The study included 60 HBV and 22 HCC patients and 40 healthy subjects. SOD, TAS, TOS, oxidative stress index (OSI), MDA, GSH, total thiol, native thiol, and disulfide levels were measured to determine serum and erythrocyte oxidant/antioxidant balance. The relationship between these biomarkers and liver function tests was evaluated.

Results

In the serum of HBV and HCC patients, significant increases were observed in MDA, TOS, and OSI levels. In contrast, significant decreases were noted in GSH, SOD, TAS, total thiol, and native thiol levels. Oxidant-antioxidant parameters in erythrocytes paralleled those in serum. In HBV patients, alanine aminotransferase and lactate dehydrogenase levels were negatively correlated with TOS and OSI. In addition, a positive correlation was found between hepatitis B surface antigen levels and total thiol and native thiol levels. MDA, SOD, and total thiol levels showed high diagnostic performance in the diagnosis of HCC with area under the curve values of 0.993, 0.800, and 0.815, respectively.

Conclusions

Measurement of serum thiol-disulfide balance and erythrocyte SOD, MDA, and TOS levels may be helpful in the diagnosis of HBV and HCC. Antioxidant support that increases GSH and thiol levels is a critical approach to prevent disease progression.

## Introduction

Hepatitis B virus (HBV) infection is recognized as an important etiological factor in the development of hepatocellular carcinoma (HCC) [1]. Chronic HBV infection leads to long-term inflammation and fibrosis in the liver, resulting in hepatocellular damage, and this process results in genetic and epigenetic changes that eventually trigger the development of HCC [2]. The main mechanisms of HBV in HCC development include direct viral oncoprotein action, chronic inflammation, and oxidative stress [3]. In this process, in which oxidative stress plays an important role, the study of antioxidant defense mechanisms involved in the protection of cellular redox balance is of critical importance in understanding the development of HBV-induced HCC [4]. Activation of antioxidant molecules such as glutathione (GSH) and enzymes such as superoxide dismutase (SOD) prevents oxidative modification of thiol groups by regulating thiol-disulfide homeostasis. Additionally, GSH is the primary thiol source in the cell. Thiol groups (-SH), which are sensitive to oxidation, can form covalent disulfide bonds (-S-S-) through redox reactions. Disulfide bonds are converted back to thiol groups by the reduction process, helping maintain cellular redox balance and mitigate oxidative stress [5]. These dynamic interactions prevent biomolecular damage, such as DNA damage, protein modification, and lipid peroxidation, and reactive oxygen species (ROS) are effectively neutralized [6].

This study aims to reveal the effects of inflammation and oxidative stress caused by chronic infection with HBV on the pathogenesis of HCC and investigate endogenous antioxidant defense mechanisms in this process. To better understand the pathophysiological mechanisms of HBV and HBV-related HCC, this study evaluated GSH, SOD, malondialdehyde (MDA), and total antioxidant/oxidant levels together with dynamic thiol-disulfide parameters. However, there is no study in the current literature that has examined thiol-disulfide balance together with GSH, total antioxidant status (TAS)-total oxidant status (TOS), oxidative stress index (OSI), SOD, and MDA parameters simultaneously at both serum and erythrocyte levels in HBV and HBV-related HCC. Most available studies have measured only serum markers or have focused on limited sub-components of the redox system. By evaluating intracellular (erythrocyte) and extracellular (serum) redox biomarkers together, our study aims to provide a more integrative biochemical picture of oxidative stress and antioxidant defense in HBV infection and its malignant transformation.

## Materials and methods

Study population

In this study, patients who applied to the Gastroenterology and Oncology Polyclinics of Kahramanmaraş Sütçü İmam University Research and Application Hospital were included. Within the scope of the study, serum and plasma samples were collected from a total of 82 patients, including 60 patients diagnosed with HBV infection and 22 patients diagnosed with HBV-related HCC. In total, 40 healthy volunteers with no prior systemic diseases were included in the control group. The study was ethically approved by the Kahramanmaraş Sütçü İmam University (KSU) Medical Research Ethics Committee (protocol number: 2024/30-268). Inclusion criteria included being between 35 and 75 years of age, being diagnosed with HBV infection, and the presence of HBV-related HCC. All HBV and HCC patients were selected from newly diagnosed individuals who had not yet started any treatment. Written informed consent was obtained from all participants.

Biochemical analysis

Blood samples were collected into gel-containing biochemistry, lithium heparin, and ethylenediaminetetraacetic acid tubes. Whole blood samples in lithium heparin tubes were centrifuged at 2,000 rpm for five minutes to separate the plasma supernatant. After centrifugation, the erythrocytes remaining in the lower phase of the tube were washed twice with saline to obtain erythrocyte packets. Blood samples taken into biochemistry tubes containing gel were centrifuged at 4,000× g for seven minutes, and the serum fraction was separated.

Measurement of serum and erythrocyte GSH, MDA, and SOD levels

Serum and erythrocyte GSH levels were determined using commercial enzyme-linked immunosorbent assay kits (Elabscience EL-0026). Serum and erythrocyte MDA levels were determined by extracting the pink-colored complex formed from the reaction with thiobarbituric acid at 95°C into the n-butanol phase [7,8]. Spectrophotometric measurements evaluated the color formation in the obtained organic phase at 520 nm and 535 nm wavelengths. MDA concentration was calculated according to the absorbance values of 1,1,3,3-tetraethoxypropane used as a standard solution. The method used for SOD measurement is based on the principle of measuring the blue formazan complex formed by the reduction of nitroblue tetrazolium (NBT) by superoxide radicals produced by xanthine oxidase. [9]. Xanthine, NBT, and xanthine oxidase were present in the reaction mixture, while the sample supernatant to be analyzed was added. In parallel, the blank was measured in microplate wells without supernatant. All samples were incubated at 25°C for 20 minutes. To terminate the reaction, copper chloride was introduced into the wells. The absorbance of the blue formazan color was measured with a Biotek 800 TS microplate reader at a wavelength of 560 nm.

Determination of serum TOS, TAS, OSI, total thiol, native thiol, and disulfide levels

Serum total antioxidant, total oxidant, native thiol, and total thiol levels were measured using Rel Assay Diagnostic colorimetric kits. Analyses were performed using the procedures described previously in detail. [8,10,11]. TAS was calculated using the formula $Result(mmol Trolox Eq/L)=\frac{&Delta;Abs_(H_2 O) - &Delta;Abs_Sample }{&Delta;Abs_(H_2 O) - &Delta;Abs_Standart }$ according to the TAS Kit procedure. TOS was calculated using the formula $Result=\frac{&Delta;Abs_Sample}{&Delta;Abs_Standart}X10$ after the procedures performed according to the TOS kit procedure. The OSI was determined by the ratio of TOS to TAS levels (TOS/TAS). In total thiol determination, after native and total thiol analysis were performed, the dynamic disulfide level was calculated using the SH / [SH + SS] ratio.

Statistical analysis

Statistical analysis was performed using GraphPad Prism version 9 (GraphPad Software, San Diego, CA, USA). Comparisons between control, HBV, and HCC groups were performed by applying the Kruskal-Wallis test for independent samples; data are presented as mean ± standard deviation (mean ± SD) in the tables. P-values were reported separately for control-HBV, control-HCC, and HBV-HCC comparisons, and a p-value <0.05 was determined as the limit of statistical significance by Dunn’s multiple comparison test. Statistical analysis for the data presented in the figures was also performed using the non-parametric Kruskal-Wallis test. The relationship between erythrocyte GSH/redox balance and serum thiol/disulfide homeostasis-related biomarkers and liver function tests was evaluated using Spearman or Pearson correlation analysis (p < 0.05 as the limit of statistical significance).

## Results

Clinical characteristics

Demographic and biochemical parameters were compared in the control (n = 40), HBV (n = 60), and HCC (n = 22) groups included in the study (Table 1).

**Table 1:** Demographic, biochemical, and hematological parameters of patients with HBV and HCC and healthy controls. The statistical differences among the control, HBV, and HCC groups were analyzed using the Kruskal-Wallis test, followed by Dunn’s post-hoc comparison for pairwise evaluations. Data are presented as mean ± standard deviation (SD) together with minimum and maximum values (Min–Max). H denotes the Kruskal-Wallis test statistic (df = 2 for three groups). A p-value <0.05 was considered statistically significant. HBV = hepatitis B virus, HCC = hepatocellular carcinoma; ALT = alanine aminotransferase; AST = aspartate aminotransferase; GGT = gamma-glutamyl transferase; ALP = alkaline phosphatase; LDH = lactate dehydrogenase; PT = prothrombin time; INR = international normalized ratio; RBC = red blood cell count; Hb = blood hemoglobin level; HCT = hematocrit; MCV = mean corpuscular volume; MCH = mean corpuscular hemoglobin; RDW = red cell distribution width; PLT = platelet count; HBsAg = hepatitis B surface antigen; AFP = alpha-fetoprotein; CEA = carcinoembryonic antigen; CA 19-9 = carbohydrate antigen 19-9

	Control		HBV		HCC		Kruskal-Wallis H (df = 2)	P-value
	Min–Max	Mean ± SD	Min–Max	Mean ± SD	Min–Max	Mean ± SD		
n	40		60		22			
Age (years)	20–66	41 ± 9	25–75	51 ± 13	52–81	65 ± 8		
ALT (U/L)	9–95	24.9 ± 17.9	7–173	28.4 ± 25.3	12–108	33.6 ± 26.3	2.252	0.32
AST (U/L)	10–53	18.6 ± 9.2	10–155	25.9 ± 23.6	17–250	67.7 ± 52.2	32.25	<0.001
GGT (U/L)	9–266	31.2 ± 53.3	9–127	33.3 ± 29.1	22–451	147 ± 120	37.57	<0.001
ALP (U/L	34–124	72 ± 20.6	43–178	84.9 ± 32.8	77–474	207 ± 105	35.93	<0.001
LDH (U/L)	133–258	180 ± 34.8	128–368	204 ± 53.9	81–816	314 ± 157	23.78	<0.001
Ferritin (µg/L)	7–205	59.9 ± 50.1	7–265	94.1 ± 70.4	9–720	240 ± 203	21.29	<0.001
Albumin (g/L)	39.5–47.2	43.5 ± 1.8	9.3–103	39.8 ± 10.0	22.5–49.2	33.1 ± 7.4	50.36	<0.001
Bilirubin (mg/dL)	0.17–1.13	0.52 ± 0.24	0.18–5.37	0.79 ± 0.83	0.23–15.7	2.71 ± 3.58	16.43	<0.001
PT (%)	67.8–123	98.5 ± 13.5	40.7–162	88.6 ± 21.8	42.2–96.2	63.6 ± 15.1	34.97	<0.001
INR	0.83–1.22	1.01 ± 0.09	0.73–109	2.97 ± 13.7	0.94–2.26	1.40 ± 0.31	28.55	<0.001
RBC (×10^6^/µL)	4.17–5.92	4.87 ± 0.43	3.32–9.70	4.69 ± 0.87	2.29–6.55	4.38 ± 1.22	5.209	0.07
Hb (g/dL) total	10.9–17.6	14.4 ± 1.39	7.3–18.7	13.7 ± 2.32	8.3–16.2	12.6 ± 2.79	4.994	0.08
HCT (%)	34.6–50.3	42.5 ± 3.58	26–51.6	40.7 ± 5.71	24.4–50.3	38.3 ± 7.84	4.107	0.13
MCV (fL)	69.1–95.5	85.6 ± 4.93	68–103	85.9 ± 6.62	75–99.2	87.3 ± 8.26	1.271	0.53
MCH (pg)	21.4–32.9	28.4 ± 2.21	19.3–33.8	28 ± 3.04	21–33.3	28.0 ± 3.36	0.037	0.98
RDW (fL)	36.3–47.8	41.0 ± 2.74	35.8–61.1	43.7 ± 5.5	37.7–68.1	52.7 ± 8.7	29.02	<0.001
PLT (×10^9^/L)	163–493	273 ± 62.7	52.4–367	216 ± 68	71.5–389	220 5 ± 99.9	16.43	<0.001
HBsAg	0.03–0.86	0.48 ± 0.14	0.19–8,276	4,445 ± 2,131	0.18–3,858	830 ± 830	75.91	<0.001
AFP (IU/mL)	0.59–6.83	2.13 ± 1.03	0.76–22.2	3.49 ± 3.58	2.48–4632	926 ± 1,316	47.63	<0.001
CEA (µg/L)	0.6–4.8	1.74 ± 0.86	0.5–11.7	2.24 ± 2.63	1.12–5.7	2.95 ± 1.45	12.52	0.002
CA 19-9 (IU/mL)	0.5–34	9.66 ± 8.0	1–48	14.1 ± 11.3	3.9–119	35.3 ± 30.1	17.25	<0.001

The control, HBV, and HCC groups had mean ages of 41 ± 9, 51 ± 13, and 65 ± 8 years, respectively. Although alanine aminotransferase (ALT) levels did not differ between the groups, aspartate aminotransferase (AST), gamma-glutamyl transferase (GGT), alkaline phosphatase (ALP), and lactate dehydrogenase (LDH) activities were significantly increased in both HBV and especially HCC groups compared to the control group (p < 0.001) (Table 2). Ferritin levels were also increased in the patient groups compared to the control group, whereas albumin concentrations were significantly decreased in both patient groups. The deterioration in total bilirubin, prothrombin time percentage (PT%), and international normalized ratio (INR) values was the most severe in HCC patients. When hematological parameters were examined, a slight decrease in erythrocyte count, hemoglobin, and hematocrit; an increase in RDW; and a reduction in platelet count were recorded. Hepatitis B surface antigen (HBsAg) levels rose significantly in the HBV group, while alpha-fetoprotein (AFP) levels were significantly higher in the HCC group than in the control and HBV groups.

**Table 2:** Dunn’s multiple comparisons (pairwise adjusted p-values) for biochemical and hematological parameters among the control, HBV, and HCC groups. This table presents adjusted p-values derived from Dunn’s post-hoc multiple comparisons following the Kruskal-Wallis analyses summarized in Table 1. Each cell corresponds to the adjusted p-value for the indicated pairwise comparison (control vs. HBV, control vs. HCC, and HBV vs. HCC). HBV = hepatitis B virus, HCC = hepatocellular carcinoma; ALT = alanine aminotransferase; AST = aspartate aminotransferase; GGT = gamma-glutamyl transferase; ALP = alkaline phosphatase; LDH = lactate dehydrogenase; PT = prothrombin time; INR = international normalized ratio; RBC = red blood cell count; Hb = blood hemoglobin level; HCT = hematocrit; MCV = mean corpuscular volume; MCH = mean corpuscular hemoglobin; RDW = red cell distribution width; PLT = platelet count; HBsAg = hepatitis B surface antigen; AFP = alpha-fetoprotein; CEA = carcinoembryonic antigen; CA 19-9 = carbohydrate antigen 19-9

Control vs. HBV		Control vs. HCC	HBV vs. HCC
ALT (U/L)	>0.99	0.41	>0.99
AST (U/L)	0.24	<0.001	<0.001
GGT (U/L)	0.19	<0.001	<0.001
ALP (U/L	0.38	<0.001	<0.001
LDH (U/L)	0.11	<0.001	0.001
Ferritin (µg/L)	0.17	<0.001	0.01
Albumin (g/L)	<0.001	<0.001	0.01
Bilirubin (mg/dL)	0.87	<0.001	0.002
PT (%)	0.02	<0.001	<0.001
INR	0.04	<0.001	<0.001
RBC (×10^6^/µL)	0.24	0.11	>0.99
Hb (g/dL) total	0.78	0.08	0.42
HCT (%)	0.26	0.02	0.22
MCV (fL)	>0.99	0.86	>0.99
MCH (pg)	>0.99	>0.99	>0.99
RDW (fL)	0.09	<0.001	<0.001
PLT (×10^9^/L)	<0.001	0.01	>0.99
HBsAg	<0.001	0.35	<0.001
AFP (IU/mL)	0.22	<0.001	<0.001
CEA (µg/L)	>0.99	0.003	0.01
CA 19-9 (IU/mL)	0.33	<0.001	0.09

Serum oxidant and antioxidant marker levels in HBV and HCC patients

**Figure 1:**
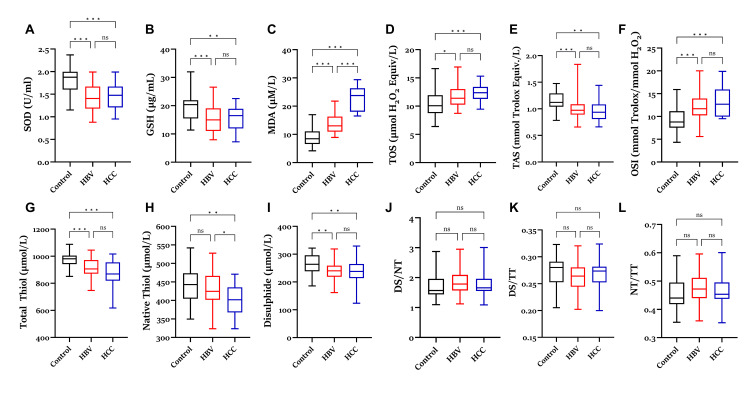
Comparison of SOD (A), GSH (B), MDA (C), TOS (D), TAS (E), OSI (F), TT (G), NT (H), DS (I), DS/NT (J), DS/TT (K), and NT/TT (L) levels in the serum of HBV and HCC patients with a control group and patient groups. *: p < 0.05, **: p < 0.01, ***: p < 0.001. SOD = superoxide dismutase; GSH = glutathione; MDA = malondialdehyde; TOS = total oxidant status; TAS = total antioxidant status; OSI = oxidative stress index; TT = total thiol; NT = native thiol; DS = disulfide; HBV = hepatitis B virus; HCC = hepatocellular carcinoma

The analysis of serum oxidant and antioxidant marker levels in HBV and HCC patients is shown in Figure 1. Among the parameters of the antioxidant defense system, in comparison with the control group, SOD and GSH levels were markedly reduced in the HBV and HCC groups. However, no significant difference was observed between the HBV and HCC groups regarding these parameters. MDA, an oxidative stress indicator, was markedly elevated in the HBV group compared to controls and showed an even higher increase in the HCC group in comparison to both the control and HBV groups. TOS levels increased in the HBV group compared to the control group, and this increase was more significant in the HCC group. TAS levels were markedly reduced in both HBV and HCC groups; however, the difference between these two groups was not statistically significant. The HBV and HCC groups exhibited significantly higher OSI levels than the control group. When the parameters related to serum thiol-disulfide balance were evaluated, total thiol and disulfide levels were significantly decreased in the HBV group, whereas total thiol, native thiol, and disulfide levels were reduced in the HCC group. Analysis revealed no significant changes in the disulfide/native thiol, disulfide/total thiol, or native thiol/total thiol ratios.

Erythrocyte oxidant and antioxidant marker levels in HBV and HCC patients

**Figure 2:**
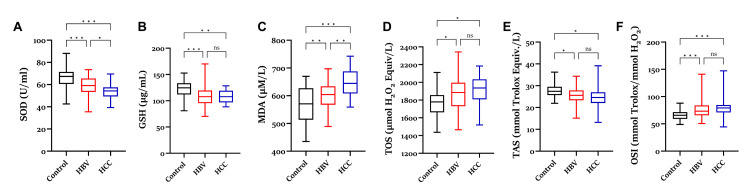
Comparison of SOD (A), GSH (B), MDA (C), TOS (D), TAS (E), and OSI (F) levels in the erythrocytes of HBV and HCC patients with a control group and patient groups. *: p < 0.05, **: p < 0.01, ***: p < 0.001. SOD = superoxide dismutase; GSH = glutathione; MDA = malondialdehyde; TOS = total oxidant status; TAS = total antioxidant status; OSI = oxidative stress index; HBV = hepatitis B virus; HCC = hepatocellular carcinoma

According to biochemical analysis obtained from erythrocyte samples, significant changes were observed in oxidative stress parameters in HBV and HCC patients (Figure 2). Both HBV and HCC groups showed a significant reduction in SOD activity compared to the control group, and this reduction was more prominent in the HCC group. Similarly, GSH levels were significantly decreased in the HBV group and were lower in the HCC group compared to the control group, but no statistically significant difference was found. MDA levels, an indicator of lipid peroxidation, showed a marked increase in both HBV and HCC groups compared to the control group. In addition, MDA levels were significantly higher in the HCC group than in the HBV group. TOS values were significantly increased in the patient groups compared to the control group, but no significant difference was found between the HBV and HCC groups. TAS levels reflecting total antioxidant capacity were significantly decreased in HBV and HCC groups compared to the control group. OSI calculated according to these data was found to be considerably higher in both HBV and HCC patients compared to the control group.

Correlation of oxidant and antioxidant biomarkers with liver function tests and tumor markers

**Figure 3:**
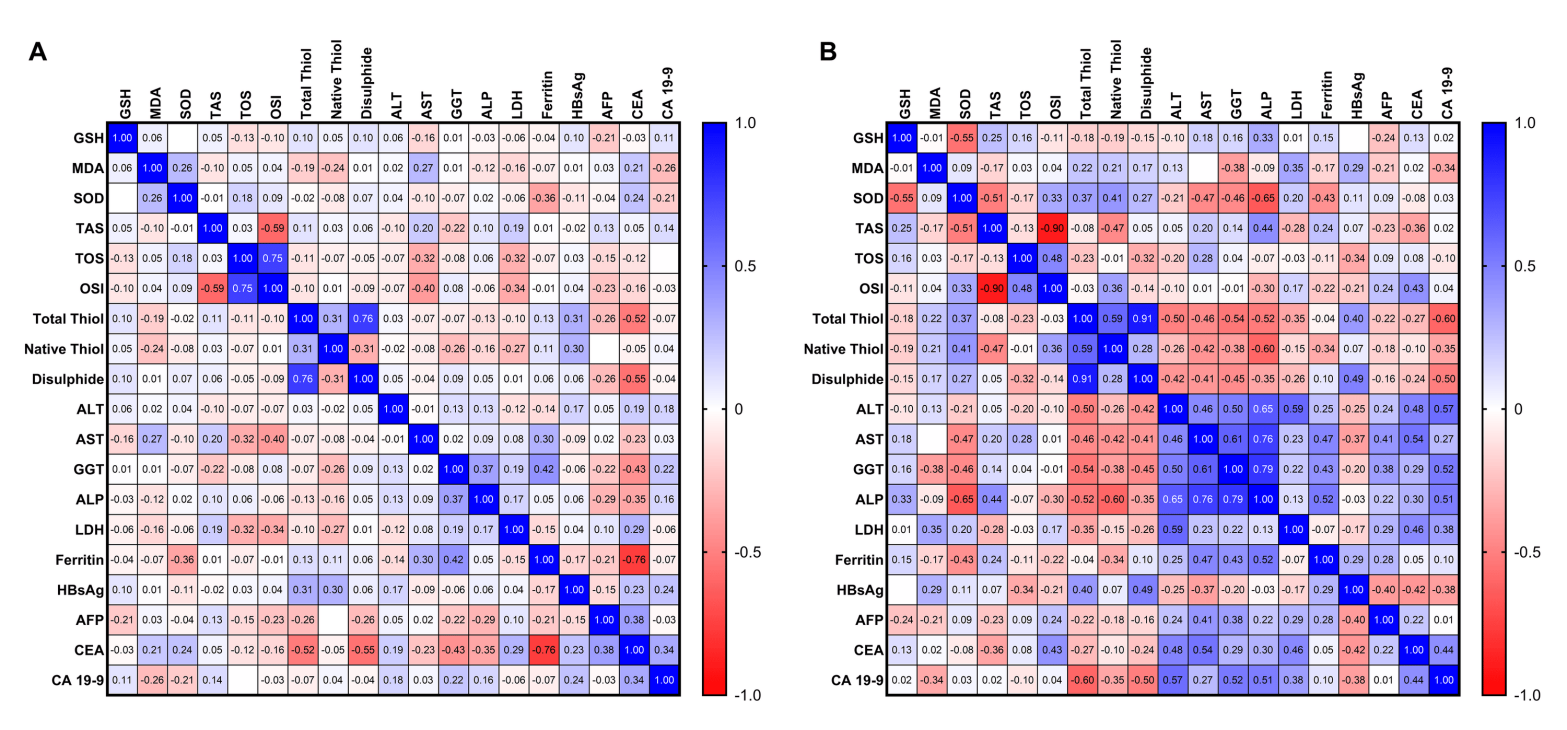
Heatmap of Spearman correlation coefficients in HBV (A) and HCC (B) patients. This heatmap visualizes the correlation coefficients (r) from Table 3 and Table 4. The color scale (shown on the right) ranges from -1.0 (strong negative correlation) to +1.0 (strong positive correlation). Shades of red represent negative correlations, whereas shades of blue represent positive correlations. The intensity of the color corresponds to the magnitude of the correlation, allowing for a quick visual assessment of the relationships among the measured parameters. SOD = superoxide dismutase; GSH = glutathione; MDA = malondialdehyde; TOS = total oxidant status; TAS = total antioxidant status; OSI = oxidative stress index; ALT = alanine aminotransferase; AST = aspartate aminotransferase; GGT = gamma-glutamyl transferase; ALP = alkaline phosphatase; LDH = lactate dehydrogenase; AFP = alpha-fetoprotein; HBsAg = hepatitis B surface antigen; CEA = carcinoembryonic antigen; CA 19-9 = carbohydrate antigen 19-9); HBV = hepatitis B virus; HCC = hepatocellular carcinoma

The correlation analysis performed in HBV patients evaluated the relationships between parameters related to oxidative stress and antioxidant defense system and liver function tests and tumor markers (Table 3, Figure 3, Panel A).

**Table 3:** Evaluation of the correlation of oxidative stress and antioxidant-related biomarkers with liver function tests and tumor markers in HBV patients. ALT = alanine aminotransferase; AST = aspartate aminotransferase; GGT = gamma-glutamyl transferase; ALP = alkaline phosphatase; LDH = lactate dehydrogenase; AFP = alpha-fetoprotein; HBsAg = hepatitis B surface antigen; CEA = carcinoembryonic antigen; CA 19-9 = carbohydrate antigen 19-9); SOD = superoxide dismutase; GSH = glutathione; MDA = malondialdehyde; TOS = total oxidant status; TAS = total antioxidant status; OSI = oxidative stress index; HBV = hepatitis B virus

		GSH	MDA	SOD	TAS	TOS	OSI	Total thiol	Native thiol	Disulfide
ALT	r	0.061	0.022	0.041	-0.099	-0.068	-0.070	0.031	-0.019	0.046
	p	0.656	0.873	0.768	0.472	0.620	0.609	0.821	0.891	0.737
AST	r	-0.163	0.274	-0.099	0.198	-0.322	-0.396	-0.075	-0.076	-0.044
	p	0.238	0.045	0.477	0.150	0.018	0.003	0.590	0.583	0.755
GGT	r	0.007	0.010	-0.069	-0.216	-0.082	0.077	-0.075	-0.260	0.085
	p	0.962	0.947	0.633	0.127	0.566	0.589	0.603	0.065	0.553
ALP	r	-0.033	-0.121	0.016	0.099	0.062	-0.056	-0.129	-0.161	0.049
	p	0.827	0.416	0.915	0.507	0.681	0.706	0.388	0.281	0.743
LDH	r	-0.058	-0.156	-0.061	0.193	-0.316	-0.336	-0.097	-0.270	0.010
	p	0.717	0.325	0.701	0.220	0.042	0.030	0.543	0.084	0.952
Ferritin	r	-0.044	-0.073	-0.360	0.007	-0.072	-0.005	0.130	0.105	0.055
	p	0.814	0.698	0.047	0.972	0.701	0.978	0.484	0.574	0.769
HBsAg	r	0.101	0.011	-0.105	-0.021	0.031	0.044	0.311	0.298	0.064
	p	0.470	0.939	0.454	0.882	0.826	0.757	0.023	0.030	0.648
AFP	r	-0.213	0.034	-0.041	0.128	-0.149	-0.232	-0.258	0,000	-0.263
	p	0.130	0.811	0.771	0.368	0.291	0.098	0.065	0.998	0.059
CEA	r	-0.026	0.212	0.235	0.045	-0.118	-0.165	-0.519	-0.051	-0.554
	p	0.923	0.410	0.360	0.863	0.650	0.525	0.034	0.845	0.023
CA 19-9	r	0.110	-0.263	-0.206	0.138	-0.005	-0.033	-0.065	0.038	-0.043
	p	0.654	0.276	0.398	0.572	0.984	0.895	0.791	0.878	0.861

A statistically significant negative correlation was found between AST levels and TOS and OSI values. Similarly, a significant negative correlation was observed between LDH levels and TOS and OSI. Ferritin levels showed a significant negative correlation only with SOD activity. Positive and significant correlations were also found between HBsAg levels and total thiol and native thiol levels. CEA levels were significantly negatively correlated with total thiol and disulfide. These findings suggest that oxidative stress markers and thiol-disulfide balance may be associated with some clinical biomarkers of liver injury in HBV patients.

In the correlation analysis performed for HCC patients, a significant negative correlation was found between ALT and total thiol (Table 4, Figure 3, Panel B). A significant negative correlation was observed between AST levels and SOD activity and total thiol. GGT values showed a significant negative correlation with SOD, total thiol, and disulfide levels. Strong and significant negative correlations were found between ALP activity and SOD, total thiol, and native thiol. The tumor marker CA 19-9 showed significant negative correlations with total thiol and disulfide levels.

**Table 4:** Evaluation of the correlation of oxidative stress and antioxidant-related biomarkers with liver function tests and tumor markers in HCC patients. ALT = alanine aminotransferase; AST = aspartate aminotransferase; GGT = gamma-glutamyl transferase; ALP = alkaline phosphatase; LDH = lactate dehydrogenase; AFP = alpha-fetoprotein; HBsAg = hepatitis B surface antigen; CEA = carcinoembryonic antigen; CA 19-9 = carbohydrate antigen 19-9); SOD = superoxide dismutase; GSH = glutathione; MDA = malondialdehyde; TOS = total oxidant status; TAS = total antioxidant status; OSI = oxidative stress index; HCC = hepatocellular carcinoma

		GSH	MDA	SOD	TAS	TOS	OSI	Total thiol	Native thiol	Disulfide
ALT	r	-0.102	0.132	-0.207	0.050	-0.196	-0.098	-0.499	-0.263	-0.417
	p	0.659	0.567	0.367	0.829	0.395	0.673	0.021	0.250	0.060
AST	r	0.179	0.001	-0.465	0.198	0.279	0.014	-0.458	-0.424	-0.410
	p	0.439	0.998	0.034	0.391	0.221	0.953	0.037	0.056	0.065
GGT	r	0.159	-0.383	-0.461	0.138	0.041	-0.010	-0.537	-0.383	-0.453
	p	0.491	0.087	0.036	0.550	0.860	0.964	0.012	0.087	0.039
ALP	r	0.326	-0.088	-0.647	0.438	-0.071	-0.296	-0.521	-0.602	-0.353
	p	0.173	0.721	0.003	0.061	0.772	0.218	0.022	0.006	0.139
LDH	r	0.008	0.347	0.205	-0.279	-0.025	0.170	-0.353	-0.152	-0.262
	p	0.975	0.134	0.387	0.233	0.916	0.474	0.127	0.522	0.265
Ferritin	r	0.153	-0.169	-0.433	0.240	-0.111	-0.220	-0.044	-0.335	0.096
	p	0.519	0.476	0.056	0.308	0.640	0.352	0.855	0.148	0.686
HBsAg	r	0.004	0.294	0.105	0.073	-0.345	-0.206	0.397	0.071	0.493
	p	0.992	0.284	0.707	0.794	0.207	0.460	0.143	0.800	0064
AFP	r	-0.240	-0.211	0.092	-0.230	0.093	0.238	-0.225	-0,181	-0.156
	p	0.294	0.360	0.693	0.316	0.689	0.298	0.327	0.432	0.500
CEA	r	0.126	0.022	-0.082	-0.360	0.082	0.429	-0.274	-0.096	-0.238
	p	0.596	0.926	0.731	0.119	0.731	0.059	0.243	0.689	0.311
CA 19-9	r	0.018	-0.341	0.033	0.016	-0.100	0.042	-0.600	-0.347	-0.504
	p	0.943	0.154	0.892	0.949	0.684	0.864	0.007	0.145	0.028

Diagnostic values of SOD, GSH, MDA, TOS, TAS, total thiol, native thiol, and Disulfide alone

**Figure 4:**
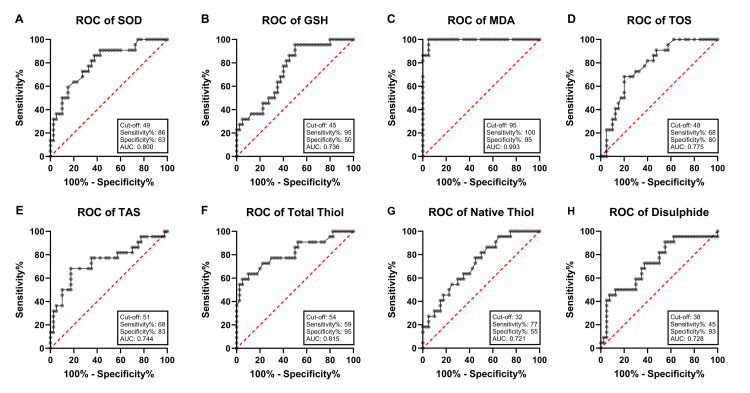
ROC analysis of SOD (A), GSH (B), MDA (C), TOS (D), TAS (E), total thiol (F), native thiol (G), and disulfide (H) biomarkers in HBV-related HCC patients. Graphs show cut-off values, AUC, and sensitivity and specificity values. SOD = superoxide dismutase; GSH = glutathione; MDA = malondialdehyde; TOS = total oxidant status; TAS = total antioxidant status; HBV = hepatitis B virus; HCC = hepatocelluar carcinoma; ROC = receiver operating characteristic; AUC = area under the curve

As a result of receiver operating characteristic curve analysis performed to evaluate the diagnostic power of oxidative stress and antioxidant parameters in HCC patients, some biomarkers were found to have high discriminative power (Figure 4). The area under the curve (AUC) value obtained for the MDA level was 0.993, the strongest discriminating parameter with 100% sensitivity and 95% specificity (cut-off = 95 µM/L). SOD activity was found to have 86% sensitivity and 63% specificity with an AUC value of 0.800, and the GSH level showed moderate diagnostic success with an AUC of 0.736, 95% sensitivity, and 50% specificity. The TOS level reflecting the oxidant status had an AUC value of 0.775 with 68% sensitivity and 80% specificity, while the AUC value for TAS was 0.744 with 68% sensitivity and 83% specificity. The discriminative power of the total thiol level was high, with an AUC value of 0.815, a sensitivity of 59%, and a specificity of 95%. Native thiol level showed moderate diagnostic accuracy with an AUC of 0.721, sensitivity of 77%, and specificity of 55%. The AUC value for the disulfide level was 0.728, and it was evaluated as a specific but limited sensitivity marker with 45% sensitivity and 93% specificity.

## Discussion

This study aimed to elucidate the pathophysiological processes leading to liver damage in HBV infection and HCC by investigating intracellular and extracellular GSH levels and endogenous antioxidant-oxidant balance. The etiopathogenesis of HBV and HCC was discussed from the perspective of redox imbalances that play a role in the disease process. In this context, to emphasize the importance of serum and erythrocyte thiol pool, GSH, total thiol, and native thiol levels, as well as TAS-TOS, MDA, and SOD levels reflecting redox balance, were evaluated.

Thiol-based enzymes and proteins are antioxidants that protect cells and tissues from oxidative damage by performing redox titration through their -SH groups. Under oxidative stress conditions, -SH groups in thiol-containing proteins show redox sensitivity and transform into disulfide bonds [12]. While thiol forms dominate under physiological conditions, increased oxidative stress disrupts the thiol/disulfide balance by increasing disulfide formation. Dynamic thiol-disulfide homeostasis serves as a crucial biochemical marker for assessing the extent of oxidative stress affecting cells and tissues [13]. Previous studies have reported significantly lower thiol levels in patients with different types of viral hepatitis compared to healthy control groups [6,14]. Dynamic thiol-disulfide homeostasis was evaluated in serum samples of patients with HBV infection and liver cirrhosis; it was reported that total and native thiol levels were significantly decreased in the patient groups compared to healthy controls, and this decrease was more pronounced in the cirrhosis group [14]. It has been shown that thiol oxidation increases, and thiol-disulfide homeostasis is disrupted in favor of the disulfide form due to increased oxidative stress in patients infected with the hepatitis C virus (HCV) [6]. In another study, it was reported that thioredoxin, one of the thiol-containing enzymes, increased in hemodialysis patients with HCV infection compared to uninfected hemodialysis patients and healthy individuals. This increase was correlated with oxidative stress levels [15]. Studies on thiol-disulfide balance in the context of viral hepatitis subtypes have mainly focused on HCV infection, and studies analyzing these parameters in HBV and HCC patients are limited. Thioredoxin expression levels were examined in HBV-associated HCC tissues and human hepatocellular carcinoma cells (HepG2) to investigate the relationship between intracellular ROS regulation and metastasis. A significant and positive relationship was noted between thioredoxin mRNA levels and HBx gene expression [16]. Another study conducted in patients with HCC and liver metastases from colorectal carcinoma investigated the expression levels of thioredoxin and glutaredoxin proteins belonging to the thiol oxidoreductase family. Immunohistochemical tissue analysis showed a significant increase in the expression levels of these proteins in both HCC and liver metastasis tissues [17]. In addition, thiol levels have been reported to be a biomarker reflecting the severity of intracellular oxidative stress in various in vitro hepatocellular carcinoma models and to participate in apoptosis signaling and enzymatic activation processes [18,19]. In this study, unlike previous reports, total thiol, native thiol, and disulfide levels were quantitatively determined in the serum of patients with HBV and HCC, and the relationships of these parameters with the clinical course were evaluated. In HBV cases, total thiol levels decreased, and a significant positive correlation was found between HBsAg levels and total thiol levels. In HCC cases, both total and native thiol levels were reduced. In addition, significant correlations were found between ALT, AST, GGT, ALP, and CA 19‑9 levels and total thiol levels.

As native and total thiol levels in plasma reflect only a limited portion of the total thiol pool, these parameters are measured together with GSH levels to more accurately assess thiol/disulfide homeostasis [5]. In addition, there are intertwined interactions of GSH, SOD, and MDA in plasma and erythrocyte redox balance [20], and determining the levels of these parameters provides essential information about systemic oxidative stress status and antioxidant defense capacity in liver diseases [21,22]. In previous studies on hepatitis, redox balance was evaluated separately in plasma and erythrocyte environments. ROS levels were analyzed in erythrocyte samples obtained from patients diagnosed with chronic hepatitis B, cirrhosis, and hepatitis B-associated acute-chronic liver failure (HB-ACLF); it was reported that oxidative stress increased significantly, especially in HB-ACLF cases [23]. In another study conducted in chronic hepatitis B cases, the relationship between oxidative stress and ALT and AST levels, which are markers of hepatocellular damage, was evaluated; a positive correlation was reported between increased serum MDA levels and ALT and AST levels [24]. In a study conducted on acute and viral hepatitis cases, it was reported that there was an increase in glutathione peroxidase (GSH-Px) levels measured in erythrocyte and plasma samples, a decrease in erythrocyte GSH levels, and an increase in erythrocyte GSH S-transferase levels [25]. In another study, erythrocyte MDA, SOD, and GSH-Px levels were evaluated in patients with acute and chronic HBV infection before and after interferon-alpha and lamivudine combination treatment. While erythrocyte MDA levels were observed to be increased before treatment in all patient groups, MDA levels were decreased after treatment, especially in the chronic HBV group. However, no significant change was reported in SOD and GSH-Px activities compared to pre-treatment [26].

In the context of HCC, it is noteworthy that there is no comprehensive study examining biomarkers reflecting the redox system simultaneously at both erythrocyte and plasma levels, and that existing studies are mostly limited to serum parameters. Levels of catalase (CAT), SOD, GSH, and MDA, which are biomarkers related to oxidative stress and the antioxidant defense system, were examined in HCC patients with and without cirrhosis and infected with HCV. In the analysis performed, an increase in MDA levels was observed in patient sera, while significant decreases in CAT, SOD, and GSH levels were reported [27]. In another study involving a total of 260 patients diagnosed with chronic hepatitis, viral hepatitis, HCC, liver cirrhosis, serum MDA, nitric oxide, GSH levels, and SOD, GSH-Px enzyme activities were evaluated. It was stated that there was an increase in oxidative stress markers and a significant decrease in antioxidant enzyme activities in patients compared to the healthy control group [28]. Unlike previous studies in the literature, in this study, oxidant (MDA, TOS, OSI) and antioxidant (SOD, GSH, TAS) biomarkers were analyzed separately quantitatively in serum and erythrocyte samples of individuals with HBV and HCC, and the correlation of these parameters with liver damage markers was evaluated. In our findings, a significant increase in serum oxidant levels and a significant decrease in antioxidant levels were detected in both HBV and HCC groups, and it was observed that these changes were more severe in the HCC group compared to HBV. A similar trend was recorded in erythrocyte samples: increased oxidant indicators and decreased antioxidant activity. In addition, the correlation of AST with TOS and OSI in the HBV group and the correlation of AST, ALP, and GGT with SOD in the HCC group are noteworthy. This suggests that TOS and OSI in HBV cases and SOD in HCC cases may be markers of hepatocellular damage. This study also evaluated the diagnostic power of oxidant and antioxidant biomarkers in HBV-associated HCC cases. MDA, total thiol, and SOD were determined to have high discriminatory power in the diagnosis of HCC with AUC values ​​of 0.993, 0.815, and 0.800, respectively. This result demonstrates the potential of MDA, total thiol, and SOD as early and reliable biomarkers of HCC.

This study has certain limitations. First, due to difficulties in patient recruitment, the number of patients included in the HCC group was relatively limited. Second, all participants were recruited from a single center, which may partially restrict the generalizability of the findings. In addition, because HCC typically develops at older ages, the HBV and HCC groups could not be age-matched, and age-related differences may have influenced oxidative stress parameters and redox biomarker profiles. Despite these limitations, the study provides valuable insights into oxidative/antioxidant status and thiol/disulfide homeostasis in patients with HBV infection and HCC, and the consistent findings observed in both serum and erythrocyte compartments support the robustness of our biochemical results.

## Conclusions

HBV and HCC pathogenesis causes changes in redox biomarkers accompanying the erythrocyte GSH system and changes the serum levels of compounds involved in the thiol cycle. This situation reveals the necessity of examining the oxidant-antioxidant system separately in erythrocytes and serum, not only in serum. Significant correlations of oxidant and antioxidant parameters with liver function tests and tumor markers indicate that these parameters may be potential indicators reflecting liver damage in HBV and HCC cases. The high sensitivity and specificity rates of MDA levels in HCC cases make it a strong diagnostic biomarker candidate. These findings show that redox biomarkers can be valuable auxiliary tools in early diagnosis and treatment monitoring, reflecting HBV pathogenesis and the transformation process to HCC. Multicenter prospective validation studies are needed to evaluate the generalizability and prognostic value of these parameters before they are introduced into clinical practice.
